# State resilience and rate of recovery among addicts: Moderating role of social skills

**DOI:** 10.3389/fpsyt.2022.906935

**Published:** 2022-08-08

**Authors:** Najam ul Hasan Abbasi, Saghir Muhammad

**Affiliations:** ^1^Department of Educational Sciences, Mianyang Normal University, Mianyang, China; ^2^Department of Psychology, International University, Islamabad, Pakistan

**Keywords:** social skills, state resilience, recovery, drug addiction, substance use

## Abstract

The present study intended to explore the moderating role of social skills in State Resilience and the rate of recovery among drug addicts. The sample size was 100 recovering addicts from different drug rehabilitation centers were recruited from twin cities (Islamabad and Rawalpindi) of Pakistan. The Social Skills Inventory (SSI) was used to assess social skills, while Connor-Davidson Resilience Scale (CD-RISC) and Substance Use Recovery Evaluator (SURE) was used to assess state resilience and recovery among study participants. Results suggest a significant positive correlation between social skills and recovery (*r* = 0.27; *p* < 0.01). Similarly, state resilience was found to be significantly correlated with social skills (*r* = 0.35; *p* < 0.01), while state resilience was not significantly correlated with recovery. The moderation analysis for the interaction of social skills on state resilience was non-significant [β = 0.002, 95% CI (−0.002, 0.00), *t* = 1.01, *p* = 0.316]. Results also indicate the significant relationship of social skills in the prevention of relapse against drug use.

## Introduction

Drug abuse is a rising concern for Pakistan in the recent past. Reports on drug use in Pakistan suggest that Pakistan has 6.7 million drug users, and more than 4 million of those numbers are addicts which is amongst the highest number for any country in the world (1). It reports that substantial portions of population aging 15–64 are suffering from overwhelming consequences of substance abuse. The report proposed that the rate of substance abuse is 5.8% which comprises of 6.4 million adults using drugs in their last 12 months and 4.1 million adults were concluded to be as dependent on drugs in Pakistan.

According to Diagnostic and statistical manual of mental disorders (2) drug addiction is a chronic and progressive illness which is linked with compulsive use of drugs and maintained by reinforcing path ways in the brain. Drug addiction is linked with biological, psychological, social and occupational impairments. Substance abuse is linked with developmental period especially with adolescence. Experimentation with drug and initiation from tobacco progress to cannabis, heroin and depressants or other psychoactive drugs and that's way tobacco use is known as a gateway to other drugs which enhances the likelihood of addictive or problematic use of drugs.

A sense of autonomy, peer pressure and idealizing negative models are associated with drug use in adolescence (3). Social skills are defined as social intelligence involving adaptive and positive social interactions (4). Social skills are linked with social intelligence and socially adaptive functioning. Furthermore, social skills are either verbal (social) or non-verbal (emotional).

Hence in present research social skills are defined and measured with six subscales of social and emotional expressivity, sensitivity and control through Social Skills Inventory (SSI) by summing up the total score of an individual. Zimmerman and Arunkumar (5) defined resilience as factors or mechanisms that inhibit the potential risks to become a full-fledged psychopathology and enhance the adaptability at the stage of adversity or generate adaptive outcomes. It is a capacity to adapt challenging or threatening circumstances and the strength to bounce back in the face of adversity (6, 7).

Hence in present study state resilience was measured by using Connor-Davidson Resilience Scale (CD-RISC) (8). So, the higher score indicated the higher state resilience in participants. Similarly, Botvin and Wills (9) found that acquisition of effective social skills is essentials for psychological adjustability and psychosocial development. So, the primary interpersonal skills are required to have confidence, responsiveness and mutually beneficial relationships as a sign of good psychosocial development but on contrary, inadequate or lack of social competence can lead to rejection and social isolation which further predicts poor psychological adjustment. The acquisition of basic social skills generally begins in childhood and it increases as individuals grow or mature with time. By the time of adolescence, they have acquired a range of social skills such as effective communication, initiation and maintenance of conversations, expression of feelings, giving and receiving of compliments, refusal of unreasonable requests. And these social skills are learned by vicarious learning and reinforcement.

Past literature on drug addiction suggests different factors which can cause drug addiction. Studies (10) showed that there are many identified risk factors which are linked with drug abuse such as peer pressure, conforming to social circles, low positive parental relationship, marital discords, intellectual inferiority, emotional immaturity, poor self (inner) control, depressive mood, violation of existing social norms. Hair et al. (11) reviewed about 360 researches of social competence among adolescents. The study stated that quality of relationships with parents is essential for the development of social competence. Moreover, social skills are also linked with psychological wellness, academic performance and interpersonal relationships. Von Hohendorff et al. (12) stated two factors individual (temperament and environment) for the development of social skills.

Resilience is a process that encompasses adaptability from crises. It is a process that helps us to cope significant adversity (13). Similarly according to (14) resilience has three occurrences such as generation of positive outcomes in vulnerable children, persistent competency during stress and recovery from trauma. They found that children who experience chronic adversity have better rate to recover completely due to the presence of positive relationship with competent person, they also have the ability to solve problems, to learn, to get along with others, and finally they have competency along with perceived self-efficacy with respect to society (15). Moreover Hiew et al. (15) stated that resilience can be differentiated in adults on the basis of its characteristics whether these characteristics are present due to current dominant states or it has been present in adults since childhood as personality trait [as cited by Bokharey (16)].

Social skills are important for interpersonal effectiveness like effectively communicating with other, reaching out, understanding other feelings and even get along with other. Similarly, sociability enhances social skills; it provides foundation for social skills. However, as per observation while working with drug addicts, it is obvious that mostly drug addicts are poor in interpersonal effectiveness and they are more likely to compensate their psychological deficits through drugs. So, it's a substantial reason that by intervening in an addict through enhancing social skills to minimize the chances of relapse. Moreover, it can help them to effectively approach their communication issues instead or drug abuse. There is no previously conducted research on social skills and resilience with respect to sociability of recovering addicts so it's unique research that aims to investigate more important social aspects which might predict relapse. This research helps in understanding the importance of social adaptability and opens new ways to intervening in addicts' life to prevent relapse.

In the present study recovery is defined as individuals who are getting outdoor treatment as follow up counseling after completing indoor treatment from drug addiction and such individuals are in the state of complete abstinence. Hence in present study recovery was measured by using Substance Use Recovery Evaluator (SURE) against five major categories such as drinking and drug use, self-care, relationships, material resources, outlook on life (17).

The current study intends to investigate the impact of social skills and state resilience on the rate of recovery among drug addicts and to further investigate the moderating effect of s social skills on state resilience among drug addicts. It was hypothesized that social skills and state resilience are positively correlated with each other and that the state resilience is negatively correlated with the use of drug among drug addicts. The study further hypothesized that social skills play a moderating role in state resilience among recovering drug addicts.

## Methods

A correlation research method was used in to find out the relationship among social skills, sociability and state resilience. The sample consisted on 100 drug addicts recruited by using purposive sampling. The age range of participants involved 3 age cohorts. These age groups were defined as late adolescents (18–24), early adulthood (24–34), and middle adulthood from (34–60) based on the Newman and Newman (18). The institutional permission was taken from International Islamic University's ethics review board before conducting the study.

### Inclusion criteria

Only recovering addicts were approached from addiction centers of Rawalpindi & Islamabad, who were discharged addicts (outdoor patients) and were receiving follow up counseling for the relapse prevention. The education level of the participants was at least under graduation (O levels, A levels and intermediate) and graduation that they could understand English as a second language.

### Exclusion criteria

The drug addicts who are still getting treatment as indoor patients and/or drug addicts who left treatment or have been discharged and do not receive any follow-up counseling were not included in the sample. Participants were selected as per English language as a second language, and participants who don't have proper education but they were in the recovery process were not enrolled in the study.

## Instruments

### Social skills inventory

Developed by Riggio (4) is a self-report measure used for assessing communication skills on two emotional (non-verbal) and social (verbal) dimensions. SSI consist six subscales of dimensions such as emotional expressivity (EE) items no. 1, 7, 13…, 85.; emotional sensitivity (ES) items no. 2, 8, 14…, 86.; emotional control (EC) items no. 3, 9, 15…, 87.; Social expressivity (SE) items no. 4, 10, 16…, 88.; social sensitivity (SS) items no. 5, 11, 17…, 89.; and social control items no. 6, 12, 18…, 90. SSI is a 5 points likert type scale continuum of “not at all like me (1), a little like me (2), like me (3), very much like me (4), exactly like me (5)”. Every subscale consists at 15 items and every sixth item belongs to same subscale and score range is from 15 to 75.

Resilience was measured through **Connor-davidson resilience scale**. CD-RISC originally developed by Connor and Davidson (8). CD-RISC has 25 items and participants rated themselves on 5-point Likert scale (0–4). Response continuum is range from not true at all = 0 to true nearly all the time strongly agree = 4. Reliability of the scale is being reported as internal consistency Cronbach's α = 0.89. Similarly, the scale also has effective convergent and discriminant validity.

### Substance use recovery evaluator

Developed by Neale et al. (17) as a valid measure for measuring recovery from drug addiction. SURE, is a 5-point Likert type scale. It comprises on 21 items. The scale also has five major categories such as drinking and drug use, self-care, relationships, material resources, outlook on life. Total Score Ranges from 21-63. The SURE is a valid measure with good face and content validity.

### Statistical analysis

The chronbach's alpha was conducted to check scales reliability, correlational analysis was conducted to investigate the relationship between study variables and moderation analysis using SPSS-22 were conducted to check the moderating effect of social skills. The level of statistical significance was set a priori at *p* < 0.05.

### Procedure

For the purpose of data collection from the participants, institutional approval was sought first. Participants were educated properly about the nature and purpose of study. After the willingness in the research and signing of informed consent they were selected for research. Instructions regarding the instruments for data collection were also given. Quarries of respondents were addressed accordingly. Participants were guided about the response pattern of questionnaires according to given instructions. In the present study, no tangible or intangible incentives were given to participants.

## Results

Table 1 indicates the alpha values for the research instruments. The calculated alpha values for Social Skills Inventory, CD-RISK and SURE (recovery) indicate that these instruments are reliable to measure social skills, state resilience and recovery.

**Table 1:** Psychometric properties of the major study variables (*N* = 100).

				**Range**			
**Variables**	***M***	***SD***	**α**	**Potential**	**Actual**	**Skew**	**Kurtosis**
1. SSKI	284.6	33.24	0.81	1–5	200–379	0.20	0.11
2. CD_RISC	73.53	18.20	0.91	0–4	28–100	−0.55	−0.66
3. SURE	51.20	8.25	0.85	1–3	28–63	−0.44	−0.46

*SSKI, Social Skills; CD_RISC, Connor-Davidson Resilience Scale; SURE, Substance Use Recovery Evaluator*.

Table 2 indicates that resilience is significantly positively correlated with SSK (*p* < 0.01). But there is a non-significant relationship between Resilience and recovery. Moreover, SSK is found to be positively correlated with Recovery (*p* < 0.01).

**Table 2:** Correlation between resilience, social skills and recovery (*N* = 100).

**Measures**	**1**	**2**	**3**
1. Resilience	1	0.35^**^	0.13
2. SSK	–	1	0.27^**^
3. Recovery	–	–	1

*SSK, Social Skills*;

** *p <0.01*.

Table 3 represents non-significant moderation present by an interaction effect, indicating that relationship between state resilience and recovery is not moderated by social skills.

**Table 3:** Linear regression using state resilience, social skills and their interaction as predictors of recovery.

	**β**	**SEβ**	***T***	***P***
Constant	50.74 (48.70, 82.79)	1.03	49.21	<0.001
Social skills	0.05 (−0.03, 0.13)	0.04	1.26	0.213
State resilience	0.03 (−0.11, 0.16)	0.07	0.42	0.677
Social skills × state resilience	0.002 (−0.002, 0.00)	0.002	1.01	0.316

*R^2^ = 0.0230*.

In order to interpret the moderation effect, simple slopes are needed to be examined as they are shown in Table 4. Basically, the table represents results of three different regressions: the regression for state resilience as a predictor of recovery (1) when social skills is low (i.e, when the value of social skills is −33.24); (2) at the mean value of social skills (when the mean value is zero); and (3) when the value of social skills is as high as 33.24. Interpretations of these three regressions are based on the value of b, and its significance.

**Table 4:** Conditional effect of state resilience on recovery at different values of social skills.

**Values of social skills**	**Effect of state resilience on *S.E* recovery (β)**	***T***	***P***
−33.24 (−1 *SD*)	−0.04, 0.07 (−0.19, 0.11)	−0.58	0.564
0.00 (*M*)	0.03 (−0.11, 0.17)	0.07	0.42
33.24 (+1 *SD*)	0.10 (−0.14, 0.34)	0.12	0.85

Three models are going to be interpreted as follows:

(1) When social skills are low, there is a non-significant positive relationship between state resilience and recovery.
(2) At the mean value of social skills, also there is a non-significant positive relationship between state resilience and recovery.
(3) When social skills is high there still remains non-significant positive relationship between state resilience and recovery.

These results tell us that the relationship between state resilience and recovery are not only determined by different levels of social skills.

**Figure 1:**
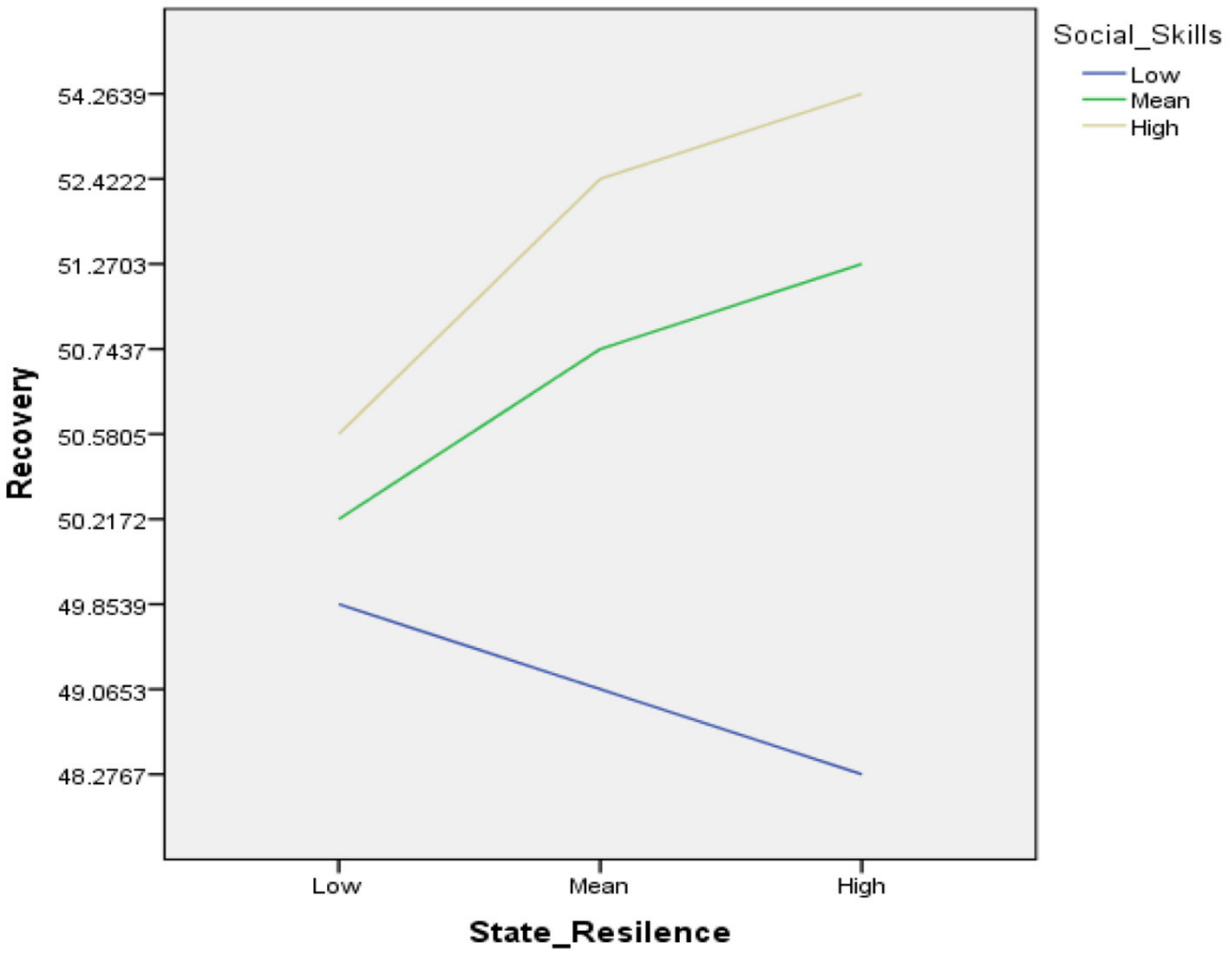
Simple slope equations of regression of state resilience on recovery at three levels of social skills.

Figure 1 shows the simple slopes analysis, graph indicates that: when the social skills is low (blue line) there is a non-significant positive relationship between state resilience and recovery; similarly, at mean and high values of social skills (green line, gray line) suggest that social skills does not significantly enhance the relationship among state resilience and recovery at both levels.

## Discussion

The present study investigated the impact of social skills and state resilience on the rate of recovery among drug addicts and whether social skills play a moderating effect on state resilience among drug addicts. It was hypothesized that social skills and state resilience are positively correlated with each other and that the state resilience is negatively correlated with the use of drug among drug addicts. The study further hypothesized that social skills play a moderating role in state resilience among recovering drug addicts.

It was hypothesized and proved that social skills are positively correlated with recovery among drug addicts. The results indicated that those individuals who were high on social skills stayed in recovery after the treatment from drug addiction. Past research showed that 11% drinkers and 50% problem drinkers having incompetent social skills while none from the non-drinkers (19).

Similarly, a meta-analysis was conducted by Ennett et al. (20) evaluated eight studies to study the effect size of drug abuse resistance education (DARE): an educational program which was taught by highly trained law enforcement officers at schools. There were six outcome measure classes such as knowledge about drugs, attitudes about drug use, social skills, self-esteem, attitude toward police, and drug use. Effect size was calculated by each outcome. Results showed that calculated effect size for social skills was significantly larger than attitudes about drug use, self-esteem, attitude toward police, and drug use. Which indicated that knowledge and social skills were helpful in intervening against drug use (20).

Another research conducted to review the 1,200 outcome studies to investigate the relationships among protective and risk factors for successful prevention programs. Findings suggested that personal and social skills, social norms, effective social policies and social support are the protective factors for preventing risks outcomes such as drug abuse, behavioral problems, school failure, AIDS, physical abuse (21). Another research showed that the social skills problems are related to more peer victimization and friendship as social factor buffer between social skills and peer victimization positively (22). Hence the results of above research also confirmed our hypothesis that social skills are positively correlated with recovery. A review conducted by Fergus and Zimmerman (23), the review of the study in which 1,184 junior high school students of new York city indicates that psychological wellbeing and social competence played a significant role of protective factors against cigarette smoking, alcohol and marijuana use.

Similarly, present study results suggest that social skills and state resilience are positively correlated. Past literature supports that social skills are essential for effective adjustment, to develop constructive interpersonal interactions. This is further associated with social, community, family and career adjustment and this social skill deficit is present in today's adolescents (24). According to the Gardner [as cited in Thompson (24)] interpersonal and intrapersonal intelligences are also associated with social skills that help in understanding others behavior, handling relationships with others, empathies with others, managing own emotions and experiences and problem solving skills. Social skills are also associated to refusal to drugs and alcohol, and saying no to premarital sex.

The results of the current studies for moderation interaction were non-significant which showed that non-significant interactions of social skills were found in state resilience among recovering drug addicts. These results were not surprising because there are few reasons. Firstly, the sample was homogeneous, 89% participants were from early and middle adulthood and there were only 3% females. Secondly, social triggers such as peers using drugs, conforming to social circles, to feel more sophisticated at socializing, to feel cool and grown up while doing drugs (25), establishing links with drug related social activities or social interactions are based on sociability. Alam et al. (10) also confirmed such social influence with drug abuse.

Moreover, according to Kring et al. (26) the social networks are linked with the use of drug and alcohol. Still, those who have vulnerability to substance use disorders are more likely to select social networks that are linked to their own drinking or drug use patterns. Social network in which a person lives is associated with individual drinking, but individual drinking also linked with more drinking of the same social network. Indeed, studies showed that effects of social selection were stronger. Social selection indicates that people are most likely to involve or choose social networks with drinking patterns like their own.

Hence in the present study participants were recovering drug addicts and according to their relapse prevention program which is comprise of disease concept, habit formation, controlled environment with supervised family intervention, they are recommended to limit their interactions with persons who use any kind of drug or to exposure to social events involving drug use. So, in recovering addicts' sociability can lead to relapse and they need to develop more careful and clean social interaction that was the reason that sociability is not playing moderation in state resilience among recovering addicts. But as we look to the conditional effect of social skills on recovery at the low level of sociability, it showed that borderline effects were established, and as we move to mean and high levels this conditional effect became non-significant.

Several studies showed that sociability play a role in continuation of cigarette smoking and drug use because the extraversion tendency to seek out such friends and peers who are in drug abuse can be a risk factor instead of a protective factor against drug abuse. Hence a study was conducted by Stein et al. (27) on initiation and maintenance of tobacco smoking in adolescence and young adulthood. They studied the sample of 461 in a cross-sectional survey at every 4 years. They assessed that smoking was positively related to extraversion, good social relationships, cigarette use of friends and cheerfulness.

Thirdly, in Pakistani culture, drug addiction is linked with bad personality or character so people are not disclosing the behavior at all. There is a stigma against drug addiction no one even imagines to be known as an addict. They have hidden places where mainly the young adults are used to visit and use drug in order to protect the prestige of their families. Even the families of drug addicts hide the matter from their relatives just because of bad name. Hence it can be justified that sociability of drug addicts is related to drug related social settings. And as per the results of the study, non-significant results related to the moderating role of sociability because during their recovery process they are under the process to develop adequate healthy social interactions as per their treatment protocol. So, there are chances that due to this process the interaction did not appeared to be significant.

### Limitations

In the present study sample was limited in number because of time constraint to conduct this research and sample also lacking the gender equality. So, in the study there were only three female participants which was not the true representative of the population. Furthermore, in the present study, most participants were from early and middle adulthood that limited the effectiveness of generalization of results. Another limitation of the study is cultural adaptation of instruments, in the present study participants were selected as per English language as a second language, and participants who don't have proper education but they were in the recovery process were not enrolled in the study. Sample of the study only comprised of 100 participants which can be increased and may show the different results.

## Conclusion

Findings of the current study indicate that there is a significantly positive relationship between social skills and state resilience. Results also show that social skills are significantly correlated with recovery from drug addiction. As per the focus of the present study, it was expected that social skills play a moderating role in state resilience among recovering addicts. But calculated results showed non-significant moderation of social skills in state resilience. These non-significant results can be justified because of homogeneous sample and cultural differences of social skills especially social skills of Pakistani papulation in relation to drug addiction.

### Implication and recommendation

Findings of the present study can be useful for the intervention strategies for preventing relapse and to enhance the rate of recovery among drug addicts. Results are showing that if we focus on social skills in drug addicts and if we apply effective management of psychosocial stressors which are linked with the state resilience of recovering addicts that will help health professionals who are currently working with drug addiction and finally it will help us to prevent the subsequent relapse from substance use. So, it will be very effective to develop some psych educational based interventions to develop awareness about drug addiction. It will also help in developing training programs to enhance students' assertiveness and social skills so that they can handle social stressors related to drug use.

In order to validate the findings of present study it is recommended that a research should be carried out on larger sample to check the moderating role of sociability. It is further recommended that equal number of distribution of male and female participants in every age cohorts can be required in order to address the gender differences. In order to establish an effective consensus among the treatment effectiveness inter-rehabs comparison is needed for the betterment of relapse prevention.

## Data availability statement

The original contributions presented in the study are included in the article/supplementary material, further inquiries can be directed to the corresponding author/s.

## Ethics statement

The studies involving human participants were reviewed and approved by International Islamic University Ethics Review Board. The patients/participants provided their written informed consent to participate in this study.

## Author contributions

NA and SM conceived of the study, collected, and analyzed the data. NA wrote and revised the paper. Both authors contributed to the article and approved the submitted version.

## Conflict of interest

The authors declare that the research was conducted in the absence of any commercial or financial relationships that could be construed as a potential conflict of interest.

## Publisher's note

All claims expressed in this article are solely those of the authors and do not necessarily represent those of their affiliated organizations, or those of the publisher, the editors and the reviewers. Any product that may be evaluated in this article, or claim that may be made by its manufacturer, is not guaranteed or endorsed by the publisher.
